# P-347. Clinical outcomes of heavily treatment-experienced people living with multidrug resistant HIV switched to a doravirine vs. non-doravirine containing regimen

**DOI:** 10.1093/ofid/ofaf695.565

**Published:** 2026-01-11

**Authors:** Charlotte-Paige M Rolle, Jamie Castano, Vu Nguyen, Federico Hinestrosa, Edwin DeJesus

**Affiliations:** Orlando Immunology Center ; Emory Rollins School of Public Health, Orlando, FL; Orlando Immunology Center, Orlando, Florida; Orlando Immunology Center, Orlando, Florida; Orlando Immunology Center, University of Central Florida College of Medicine, Orlando, FL; Orlando Immunology Center, University of Central Florida College of Medicine, Orlando, FL

## Abstract

**Background:**

Recent studies suggest that doravirine (DOR) may have a role in the management of heavily treatment-experienced (HTE) people with HIV (PWH), however, further data is needed to validate these findings. Here, we present clinical outcomes of viremic and aviremic HTE multidrug resistant (MDR) PWH switched to a DOR vs. non-DOR containing regimen through 48 weeks.

**Figure:**
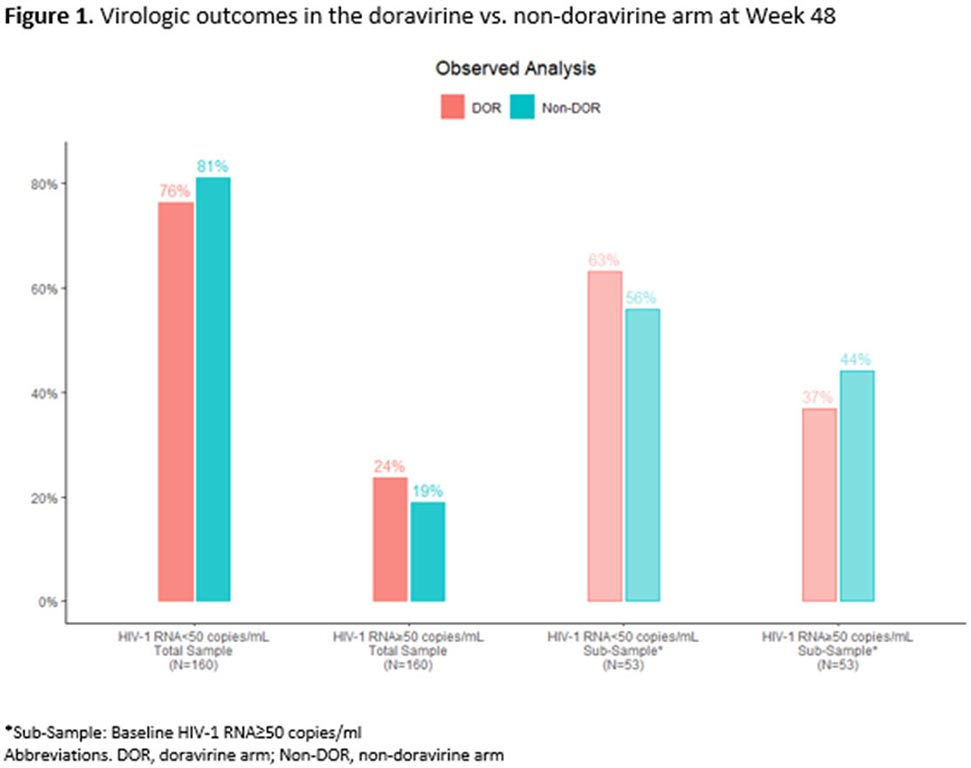

**Methods:**

This observational, single-center study evaluated medical records from viremic and aviremic PWH with resistance to ≥2 antiretroviral classes who were switched to a DOR vs. non-DOR containing regimen between 9/1/2018 and 5/1/2023. Primary endpoint was the proportion with HIV-1 RNA< 50 c/mL at Week 48 following switch. Safety and changes in renal and metabolic parameters were also evaluated.

**Results:**

167 were enrolled (24% women, mean age 51 years, 54% non-white), 45 switched to a DOR-containing regimen and 122 switched to a non-DOR containing regimen. Mean baseline HIV-1 RNA was 49,893 c/mL in the DOR arm vs. 11,927 c/mL in the non-DOR arm. Mean combined genotypic sensitivity score (cGSS) was 2.5 in the DOR arm vs. 2.3 in the non-DOR arm and mean number of fully active drugs in the regimen was 2.1 in both arms. At Week 48, among those with available data, 29/38 (76%) in the DOR arm and 99/122 (81%) in the non-DOR arm had HIV-1 RNA< 50 c/mL (p=0.26). Among those viremic at Week 48, 1/9 in the DOR arm and 7/23 in the non-DOR arm had post-switch genotypes, 1 in the DOR arm and 0 in the non-DOR arm had new resistance. Of those with baseline HIV-1 RNA≥50 c/mL, 12/19 (63%) in the DOR arm vs. 19/34 (56%) in the non-DOR arm achieved an HIV-1 RNA< 50 c/mL at Week 48 (p=0.42). The proportion with HIV-1 RNA< 50 c/mL did not differ by cGSS or number of fully active drugs in the regimen in either arm at Week 48. There were no significant differences in drug-related AEs and discontinuations, or change from baseline in renal, lipid or metabolic parameters between arms at Week 48.

**Conclusion:**

In HTE MDR PWH, switching to a DOR- and non-DOR containing regimen were both associated with achievement and maintenance of viral suppression in similar proportions regardless of baseline HIV-1 RNA, cGSS and number of fully active drugs in the regimen. These data support the potential use of DOR in MDR PWH unable to achieve or sustain viral suppression due to limited treatment options.

**Disclosures:**

Charlotte-Paige M. Rolle, MD, MPH, Gilead Sciences: Grant/Research Support|Gilead Sciences: Honoraria|MSD: Grant/Research Support|ViiV Healthcare: Advisor/Consultant|ViiV Healthcare: Grant/Research Support|ViiV Healthcare: Honoraria Federico Hinestrosa, MD, Gilead Sciences: Honoraria|MSD: Honoraria

